# *Candida krusei* Empyema: A Lung Transplant Case and Systematic Review of the Literature

**DOI:** 10.3390/jof11100735

**Published:** 2025-10-13

**Authors:** Shifa Karatela, Sangeeta Nair-Collins, Gabriel Godart, Mary Ann Peacock, Kelly Larimore, Kristin Cuthbert, Bala Munipalli, Rohit Chitale, Ravi Durvasula, Justin Oring

**Affiliations:** 1 Division of Infectious Diseases, Mayo Clinic, Jacksonville, FL 32224, USA; karatelashifa19@gmail.com (S.K.); godart.gabriel@mayo.edu (G.G.); peacock.maryann@mayo.edu (M.A.P.); chitale.rohit@mayo.edu (R.C.);; 2 Department of Internal Medicine, Mayo Clinic, Jacksonville, FL 32224, USA

**Keywords:** *Candida krusei*, empyema, biofilm, *Pichia kudriavzevii*, invasive candidiasis

## Abstract

*Candida krusei* empyema is a rare but serious manifestation of invasive candidiasis, characterized by intrinsic resistance to fluconazole, biofilm formation, and high mortality, with limited case-level data to inform management. This review aims to systematically identify and synthesize all reported English-language cases of *Candida krusei* empyema from January 2005 to June 2025 using PubMed, ScienceDirect, OVID MEDLINE, and Gale OneFile and perform descriptive analysis on them. Screening, data extraction, and eligibility assessment were performed, and those articles not clearly meeting eligibility criteria were reviewed by additional reviewers with consensus resolution. Seven publications (six individual cases and two cohorts) were included. We additionally describe the clinical course, management, and outcome of a 70-year-old bilateral lung transplant patient who developed persistent *C. krusei* empyema despite optimized antifungal therapy. Patients ranged from 11 to 74 years of age (median 62.5 years). Predisposing factors included esophageal perforation (n = 4), post-transplant hemorrhage (n = 1), community-acquired empyema (n = 1), and thoracic surgery (n = 1). Empiric fluconazole was switched to caspofungin (3/4), with others receiving amphotericin B, voriconazole, or combination therapy. Source control varied: chest tube drainage (n = 3), percutaneous catheter (n = 3), and surgical decortication (n = 2). Mortality was 14.3% (1/7). In the absence of clear guidelines and robust literature, the management approach remains heterogeneous. Optimal care requires early recognition, aggressive multimodal antifungal therapy, and effective source control tailored to patient risk. Standardized antifungal protocols and larger case series are needed to guide clinicians in managing this challenging infection.

## 1. Introduction

Although *Candida albicans* is the most common cause of candidiasis, infections due to *non-albicans Candida* (NAC) species, like *Candida krusei*, have been increasingly reported [1]. In the United States, candidemia-related hospitalizations rose by 52% between 2000 and 2005, from 3.65 to 5.56 cases per 100,000 population, and by 49% per 1000 hospitalizations, from 0.28 to 0.42 cases [2]. While a growing number of Candida species are isolated from patients with invasive candidiasis (IC) and candidemia, over 90% of cases are attributed to five species: *C. albicans*, *C. glabrata*, *C. tropicalis*, *C. parapsilosis*, and *C. krusei* [3]. Among these, *C. krusei* is the least frequently isolated and is strongly associated with severe immunosuppression and high mortality rates, with reported case fatality rates approaching 49% [4].

In these immunocompromised patients, Candida infections often arise early post-transplant due to factors such as indwelling catheters, surgical complications, or bronchial anastomotic dehiscence [5]. Although invasive pulmonary candidiasis is less frequent than mold infections, Candida can still infect the airways; for example, fungal colonization or infection at the bronchial anastomosis site has been reported in approximately 3–10% of lung transplant cases [6]. *C. krusei* is a notable *non-albicans species* in this setting, known for its intrinsic resistance to fluconazole. This species can colonize the respiratory tract and form adherent biofilms on mucosal surfaces or prosthetic material (like airway stents), further promoting persistent infection in an immunosuppressed host [5,7].

*C. krusei*, now known as Pichia kudriavzevii [8], has several reported cases of infection involving the bloodstream, eyes, joints, and endocardium in immunocompromised or hospitalized patients [9]. However, involvement of pleural space infections, empyema, remains exceedingly rare.

Pleural infections such as empyema caused by *C. krusei* are particularly challenging due to the organism’s intrinsic resistance to fluconazole and reduced susceptibility to other azoles. The pathogen’s ability to form biofilms further complicates treatment by impeding antifungal penetration, shielding it from host immune responses, and facilitating the development of resistance. In immunocompromised patients, these biofilms contribute to deep tissue invasion, immune evasion, and extensive tissue damage [10].

To date, *C. krusei* empyema remains poorly characterized, with only seven articles reported in the literature, including our case in a post-lung transplant patient. Despite the vulnerability of solid organ transplant recipients, especially lung transplant patients, due to prolonged immunosuppression and exposure to broad-spectrum antimicrobials, reports in this population are exceedingly rare. This knowledge gap hampers the development of evidence-based diagnostic and therapeutic strategies for this high-risk group.

In this review, we aim to conduct a systematic search and synthesize all reported cases of *Candida krusei* empyema. Additionally, we present a rare case in a lung transplant recipient, providing insights into its diagnosis, management, and clinical implications.

## 2. Materials and Methods

We conducted a comprehensive search on 7 June 2025, across four major biomedical databases, to identify all reports of *Candida krusei* empyema. The same core search syntax was adapted to each platform. Data were extracted into Microsoft Excel, and descriptive analyses were performed; any missing data were left blank and excluded from the relevant analyses.

### 2.1. Search Strategy

A comprehensive search of the available literature was completed in PubMed, ScienceDirect, OVID MEDLINE, and Gale OneFile: Health and Medicine databases. The search results were limited to English-language journal publications from January 2005 through June 2025 (the last 20 years). Search terms were developed to focus on a descriptive analysis of the presentation, treatment, and outcomes (O) of patients with *Candida krusei* pleural empyema (P) caused by *Candida krusei* (I).

We combined free-text terms (“*Candida krusei*” OR “*Pichia kudriavzevii*”) with empyema-related keywords (“empyema”, “pleural empyema”, “pulmonary empyema”, “thoracic empyema”, “empyema chest”, “pyothorax”) and MeSH terms (“Empyema, Pleural”, “Empyema”). Table 1 provides a summary of the database-level search strategy.

These eight database-level inclusions correspond to seven unique publications due to overlap across sources.

### 2.2. Eligibility Criteria

Studies were eligible for inclusion if they described human cases of pleural empyema caused by *Candida krusei* (*Pichia kudriavzevii*), confirmed through culture or equivalent microbiological methods, and reported relevant clinical data on presentation, underlying risk factors, management strategies, antifungal therapy, or outcomes. Case reports, case series, and cohort studies providing either patient-level or cohort-level data were included, limited to English-language publications between January 2005 and June 2025. Exclusion criteria comprised studies reporting empyema due to non-*C. krusei* species, *C. krusei* infections in non-pleural sites, articles without original patient data (e.g., reviews, abstracts, commentaries), non-human studies, and reports lacking species-level identification.

### 2.3. Study Selection

**Figure 1:**
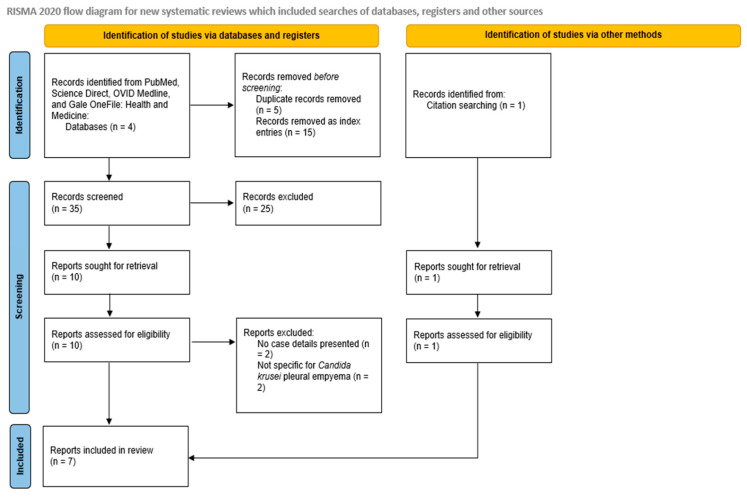
PRISMA flow diagram.

The initial search yielded 55 records. After removing 5 duplicates, 50 unique records underwent title and abstract screening. Of these, 15 index entries and 25 non-relevant articles (20 not pleural empyema and 5 not *C. krusei* pleural empyema) were excluded. A full-text review of 10 articles resulted in the exclusion of 2 studies lacking individual case data and 2 studies that were not specific to *Candida krusei* pleural empyema. Citation tracking identified one additional case. Ultimately, seven unique publications met the inclusion criteria (six from database searches plus one via citation tracking). A PRISMA flow diagram illustrates this process (Figure 1). All screening, eligibility, and data extraction steps were independently performed by K.C. and verified by K.L. and M.A.P. The review was conducted in accordance with PRISMA 2020 guidelines.

## 3. Case Report

A 70-year-old man with idiopathic pulmonary fibrosis (IPF) and exertional hypoxia was admitted to a tertiary care center in Florida, USA, for bilateral lung transplantation on 29 July 2024. His pretransplant course included evaluation for pulmonary hypertension (normal right heart hemodynamics on 12 June 2024) and a left sinus work-up to rule out infection. He received basiliximab induction, then maintenance immunosuppression with tacrolimus (Prograf), mycophenolate (CellCept), and a prednisone taper. Prophylaxis included trimethoprim-sulfamethoxazole (PJP), valganciclovir (CMV/HSV), and routine vaccinations.

On the day of transplant (29 July 2024), bronchoalveolar lavage (BAL) and bronchial tissue grew *Candida krusei*. Caspofungin was started immediately but discontinued the next day due to transaminitis; anidulafungin was given for 14 days, then stopped when transaminases normalized. Despite this, serial BALs on 1 August and 8 August remained positive for *C. krusei*. On 7 August, therapy was switched to voriconazole 200 mg PO BID (with daily QT monitoring because of concurrent amiodarone), and intrapulmonary amphotericin B was added on 3 December 2024 when loculated collections persisted. Voriconazole dosing was increased (300 mg PO BID) on 26 August once *C. meyerozyma* appeared alongside *C. krusei*, and therapeutic trough levels (4.2 µg/mL on 30 August; 2.4 µg/mL on 4 November) were documented. By late October, a right thoracentesis (16 October) and pigtail catheter placement (17 October) confirmed an exudative, loculated empyema with *C. krusei* susceptible to voriconazole, micafungin, and anidulafungin. Caspofungin was resumed briefly on 17 October, then replaced by oral voriconazole 200 mg twice daily on 29 October; itraconazole prophylaxis was planned after six weeks of voriconazole. A right chest tube was placed on 5 November after recurrent pleural effusion, again yielding *C. krusei* despite ongoing voriconazole.

Throughout November and December 2024, the patient remained on voriconazole (200 mg twice daily) with intrapleural amphotericin B instillation on 3 December 2024; chest tubes were removed on 27 December after a slow resolution. Hypogammaglobulinemia prompted IVIG in early January 2025. Voriconazole was discontinued on 19 February 2025 after more than eight weeks of therapy, with plans for close monitoring. However, on 7 March 2025, he re-presented with a drop in spirometry and a recurrent left pleural effusion; thoracentesis again grew *C. krusei* (despite therapeutic voriconazole levels, last measured at 3.4 µg/mL on 2 December 2024). Dual therapy with voriconazole 300 mg BID plus micafungin 100 mg daily was initiated on 8 March 2025, and a chest tube drained 400 cc. Persistent empyema was attributed to a biofilm-laden pleural rind; cardiothoracic surgery evaluation for possible decortication was recommended, but prolonged antifungal therapy (6–12 months of voriconazole, with possible lifelong suppression) was chosen given his immunosuppression. In mid-March, he also developed a marginal zone lymphoma (RUL nodule on 3 April 2025), and his voriconazole trough was subtherapeutic (0.4 µg/mL), prompting dose adjustments. By early April, BAL and pleural fluid cultures had turned negative, and he continued voriconazole 250 mg BID alongside IV vancomycin for a concomitant Staph. epidermidis empyema. As of 9 April 2025, he remained clinically stable, with slow radiographic improvement of the loculated empyema, and plans to continue voriconazole through at least January 2026, balancing the risks of persistent biofilm versus surgical intervention.

## 4. Results

**Figure 2:**
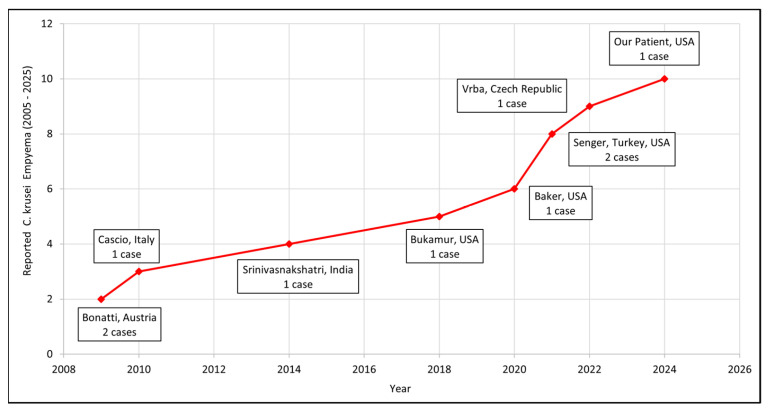
Trend in reported *Candida krusei* empyema cases over the last 20 years (2005–2025).

From the seven included studies, we identified nine published cases of *C. krusei* empyema, and with the addition of our patient, the total reached ten. Of these, five studies provided individual patient-level data [11,12,13,14,15], accounting for six patients; two presented cohort-level data (Baker et al. [16] and Senger et al. [17]). Baker et al. reported five episodes of *C. krusei* infection within a lung transplant cohort (n = 815), of which one involved the pleural space [16]. Senger et al., in a multicenter retrospective study of 81 patients with Candida empyema, found *C. krusei* in 2% (2/81) of cases [17]. Table 2 summarizes the clinical characteristics, management strategies, antifungal regimens, and outcomes of patients with available individual-level data. Detailed patient-level information was not available for the three cases described by Baker et al. and Senger et al., and thus, these could not be included in the descriptive analysis. Figure 2 depicts the trend in reported cases over the last two decades.

The patients ranged in age from 11 to 74 years (median: 62.5 years) and included three males and four females. Predisposing settings were most commonly esophageal perforation (n = 4), including three cases of Boerhaave syndrome and one postoperative anastomotic dehiscence. Other contexts included post-lung transplant hemorrhage (n = 1), community-acquired empyema (n = 1), and post-lung transplant thoracic surgery in our patient.

Clinical presentations were diverse; Srinivasnakshatri et al. reported abdominal pain, nausea, and dyspnea (SpO_2_ 90–92%) [13]. Bukamur et al. described acute dyspnea, hypotension, and cyanosis in a 74-year-old woman with schizophrenia and COPD [14]. Cascio et al. detailed fever, fatigue, dyspnea, and nausea in a pregnant woman with hydropneumothorax [12]. Vrba et al. described high fevers and bilateral fluidothoraces in a postoperative esophageal carcinoma patient [15].

Empiric fluconazole was initiated in four of seven cases, later modified to caspofungin in three, and continued in one. The remaining three patients were newly started on amphotericin B (n = 1), voriconazole (n = 1), or a combination of voriconazole and micafungin (n = 1). Source control strategies included chest-tube drainage (n = 3), percutaneous catheter placement (n = 3), and surgical decortication (n = 2). In-hospital mortality was 14.3% (1/7); two additional patients later died of unrelated causes, while four survived without documented long-term complications.

## 5. Discussion

*Candida krusei* empyema is an uncommon manifestation of invasive candidiasis, affecting immunocompromised, critically ill, and post-surgical patients [18]. In lung transplant recipients on immunosuppression, invasive candidiasis is a common and serious complication. *Candida krusei*, a notable *non-albicans species* in this setting, frequently colonizes the respiratory tract and forms biofilms that adhere to both abiotic surfaces (such as airway stents and tubes) and biotic surfaces (such as mucosa and airway tissues) [5,7].

Although compromised immunity is a well-recognized risk factor for systemic fungal infections, invasive candidiasis may also occur in patients without an evident underlying immunodeficiency, as was observed in five of the seven reviewed cases [19]. The remaining two patients were immunosuppressed, having received basiliximab induction followed by maintenance therapy with tacrolimus, mycophenolate, and a prednisone taper in the setting of lung transplantation. Esophageal perforation or anastomotic dehiscence was the most reported underlying condition in cases of *C. krusei* empyema (n = 4), followed by post-lung transplant (n = 2) and post-PDA device closure (n = 1). A unifying feature across these cases was prior chest manipulation or exposure of the lungs to gastrointestinal pathogens. It usually occurs following recent thoracic surgery, esophageal rupture, or chest instrumentation, which acts as the portal of entry for the fungus in the host. Cascio et al. emphasized this association, noting that the presence of fungal empyema should raise clinical suspicion for an underlying esophageal rupture [12]. Once the entry is accessed, the fungus then adheres to the host tissue by forming biofilm on pleural membranes and indwelling chest tubes, creating a persistent focus of infection.

*Candida krusei* was consistently identified through culture-based methods from relevant clinical specimens. Bonatti et al. isolated the organism from pleural fluid obtained by pigtail catheter drainage in a 58-year-old female and from a mediastinal abscess in a 59-year-old male [11]. Cascio et al. reported characteristic colony morphology on blood agar and Sabouraud dextrose agar, with subsequent microscopic and biochemical confirmation, including demonstration of intrinsic fluconazole resistance [12]. Srinivasnakshatri et al. demonstrated growth of *C. krusei* alongside *C. tropicalis* on CHROMagar following diagnostic thoracentesis [13]. In the case described by Bukamur et al., *C. krusei* was repeatedly recovered from pleural and sputum samples [14]. Vrba et al. documented isolation of the fungus from multiple sites, including pleural empyema fluid, sputum, blood, and the disrupted esophageal anastomosis during ICU stay [15]. In our case, *C. krusei* was repeatedly isolated from BAL, bronchial tissue, and thoracocentesis fluid.

In all cases, antifungal susceptibility testing (AFST) was conducted to establish the drug resistance profile of *C. krusei* and guide definitive therapy. Although fluconazole is intrinsically ineffective against this species, it was occasionally administered as prophylaxis [11] or empirically [12] in critically ill or post-transplant patients. Once AFST results became available, management was appropriately modified, with echinocandins or voriconazole employed as targeted treatment.

In a multicenter study of 81 patients with Candida empyema, *Candida krusei* accounted for 2% of cases. These patients had significant morbidity: more than 80% required intensive care, and approximately 27% died within 100 days despite treatment [17]. In our review, mortality directly attributable to *C. krusei* infection was 1/7 (14.3%), whereas the all-cause mortality rate was 3/7 (42.8%), suggesting that deaths were more often related to severe comorbidities and complications rather than the infection itself. Nguyen et al. identified neutropenia, lymphoma, prior glucocorticoid use, chronic liver disease, and elevated creatinine as predictors of mortality in *C. krusei* infection [4].

The mortality associated with Candida empyema may in part be explained by its biofilm-forming ability, which makes eradication challenging. Biofilms provide a protected niche with an extracellular matrix that shields fungal cells from antifungal agents and host immune responses [7]. This environment also facilitates bacterial co-infections, which are reported in more than half of Candida empyema cases, particularly among immunosuppressed and critically ill patients [6]. Furthermore, biofilm-associated Candida can tolerate high concentrations of antifungal drugs through multidrug resistance mechanisms such as upregulated efflux pumps and stress-responsive pathways [5]. This biofilm-mediated persistence promotes inflammation, tissue damage, and resistance to antifungal therapy, which can lead to chronic colonization and complications such as bronchial anastomosis dehiscence, graft dysfunction, and delayed healing. It is difficult to differentiate mere colonization from true invasive Candida infection. Hence, when *C. krusei* is involved, with its ability to form biofilm and its intrinsic resistance to fluconazole, it is wise to treat it aggressively with an echinocandin rather than an azole and thorough debridement of tissue to achieve microbiological cure and prevent relapse [6].

Because of these difficulties, aggressive, multimodal management is necessary to achieve positive results in cases of Candida empyema. It is imperative to diagnose Candida empyema as soon as possible and use treatment that includes prompt antifungal initiation, drainage techniques, and the removal of infected material [17]. Patients who received both sufficient source control and efficient antifungal treatment fared better. Candida empyema, however, has a high mortality rate and frequently necessitates lengthy antifungal courses, even with the best care [5,7]. Improved antifungal techniques, like combination therapy or new agents, are being investigated to break through Candida biofilms and lessen the need for invasive surgical procedures [6].

### 5.1. Treatment Challenges and Lacunae in Guidelines

The American Society of Transplantation (AST) Infectious Diseases Community of Practice recommends that in lung transplant recipients, Candida isolated from lower respiratory tract cultures usually represents colonization and should not be treated. Treatment is indicated only for high quantitative growth (≥10^5^ CFU/mL) in donor or recipient BAL at transplant or for ulcerative tracheobronchitis. The risk of invasive candidiasis is highest within 30 days post-transplant. For invasive disease, therapy with an echinocandin or fluconazole for 2 weeks is recommended, tailored to the organism’s susceptibility [20].

The European Society of Clinical Microbiology and Infectious Diseases (ESCMID) similarly emphasizes that Candida isolation from respiratory secretions should not trigger treatment, as it usually reflects colonization, though it recommends antifungal therapy for proven invasive candidiasis and in ICU patients with Candida detected in respiratory samples. The role of serum/plasma (1,3)-β-D-glucan to guide antifungal therapy is only marginally supported [21].

The European Confederation of Medical Mycology (ECMM), in cooperation with the International Society for Human and Animal Mycology (ISHAM) and the American Society for Microbiology (ASM), recently issued a guideline for Candida empyema thoracis based on four studies encompassing a total of 366 patients [19]. The recommendations include pleural drainage and surgery (SoR = A, QoE = III), fluconazole therapy (SoR = B, QoE = III), and azoles for cancer patients (SoR = A, QoE = III). These preferences are explained by pharmacokinetic observations: echinocandin concentrations in pleural effusion (as well as in peritoneal fluid and wound secretions) are significantly lower than in plasma, raising concerns about treatment failure and resistance development. In cases of echinocandin treatment failure, dose escalation or switching to another antifungal class is advised. Diagnostic evaluation and source control through pleural drainage or thoracoscopic decortication are strongly recommended. Since *C. krusei* is intrinsically resistant to fluconazole, echinocandins remain the preferred treatment despite their limited pleural penetration, making meticulous source control essential for achieving a cure.

None of these guidelines provides specific treatment regimens for pleural candidiasis, Candida empyema, or therapy tailored to *Candida krusei*. They also lack recommendations on optimal antifungal duration, the role of combination therapy, and standardized strategies for integrating surgical source control with antifungal treatment.

This scant and heterogeneous literature contrasts sharply with the complexity of our case: a recent bilateral lung transplant recipient with persistent *C. krusei* empyema despite optimized antifungal therapy. In such cases, *C. krusei* detected in BAL and bronchial tissue immediately post-transplant could technically be interpreted as colonization. However, in the context of a bilateral lung transplant, repeated recovery of a fluconazole-resistant organism, and the risk of biofilm formation at the bronchial anastomoses, antifungal therapy was initiated early. Radiographic evidence of pleural empyema only emerged on day eighty-one, which could be considered the true onset of invasive disease. In other words, the early cultures reflected an evolving invasive process that later manifested clinically as empyema. This illustrates the difficulty of distinguishing colonization from infection in immunosuppressed lung transplant recipients.

Although surgical decortication or VATS may provide improved source control in refractory cases, these procedures carry substantial risks in newly transplanted lungs, and transplant surgeons may be reluctant to intervene surgically in the immediate post-transplant period. Our patient was initially managed with single-agent caspofungin and subsequently with voriconazole monotherapy. Dual antifungal therapy (voriconazole plus micafungin) was reserved for a brief, targeted period during recurrence, supplemented by intrapleural amphotericin B. Given the heightened risks of surgical intervention so soon after transplant, we deferred decortication, opting instead for extended medical therapy with close monitoring and readiness to reconsider surgery if necessary.

### 5.2. Recent Pharmacological Developments

Several innovative pharmacological approaches are being developed and making their way from the lab to the patient’s bedside. For example, experimental compounds such as T-2307 and PQA-18 have demonstrated significant activity against biofilm-forming Candida species, including eradication of mature biomass and reduction of fungal burden in preclinical models [22]. Repurposing known drugs has also shown promise: Niclosamide, originally an antihelminthic, disrupts biofilm structure and filamentation, and when reformulated as nanoparticles, clears mucosal candidiasis caused by fluconazole-resistant strains in mice [23].

Beyond pharmacologics, delivery innovations play a crucial role. Nanoparticles, liposomes, and encochleated formulations improve drug penetration into biofilms and reduce systemic toxicity [24]. One up-and-coming discovery is mandimycin, a glycosylated macrolide antibiotic with a novel mechanism that targets membrane phospholipids, showing potent activity against resistant fungal strains and robust efficacy in animal models [25]. These efforts suggest that translational antifungal research is not only active but increasingly nuanced, targeting both fungal biology and drug delivery barriers. As such, innovations that advance toward clinical integration hold the potential to transform outcomes for patients with resistant biofilm-related fungal infections.

In this review, we highlight two major challenges in the management of *Candida krusei* empyema: the lack of standardized evidence-based guidelines and treatment protocols, and the difficulty of balancing aggressive source control with the risks inherent to recent lung transplantation. The management in cases like ours would continue to depend heavily on multidisciplinary judgment, individualized risk-benefit assessment for surgical intervention, close monitoring of therapeutic drug levels, and clinical response until guidelines emerge with larger case series.

Future research should aim to consolidate case-level data, define optimal antifungal combinations and durations, and clarify the role and timing of surgical procedures such as VATS or decortication in immunocompromised hosts.

### 5.3. Limitations

To date, only seven articles have been published, documenting a total of thirteen cases of *Candida krusei* empyema in the literature. Drawing conclusions or identifying trends from such a small data set is inherently challenging and often lacks statistical significance. The limited sample size restricts the robustness and generalizability of our findings. Not all cases of *C. krusei* empyema have likely been published, possibly due to diagnostic challenges, lack of awareness, or reporting bias. This underrepresentation means the current literature does not accurately reflect the true prevalence of the disease, potentially skewing the perceived clinical spectrum and outcomes. The cases reported so far arise from a narrow demographic and geographic spread. Given that *C. krusei* may exhibit regional variability in pathogenicity, resistance patterns, and clinical presentation, the generalizability of our conclusions to broader or more diverse populations is limited.

## 6. Conclusions

Empyema caused by *Candida krusei* is an uncommon but severe manifestation of invasive fungal disease, associated with significant treatment challenges due to its natural resistance to fluconazole, ability to form biofilms, and high fatality rates. Given the scarcity of reported cases, there is no consensus on optimal treatment, and current approaches vary widely. Favorable outcomes appear to rely on early detection, individualized antifungal regimens, often involving drug combinations, and timely, effective source control. There is a clear need for larger datasets and standardized treatment strategies to better inform clinical practice for this rare but critical condition.

## Figures and Tables

**Table 1:** Search Summary.

Database	Date of Search	Records Retrieved	Included Studies
PubMed	7 June 2025	5	2
ScienceDirect	7 June 2025	45	2
OVID MEDLINE	7 June 2025	2	2
Gale OneFile: Health and Medicine	7 June 2025	3	2

**Table 2:** Summary of published cases of *Candida krusei* empyema.

First Author, Year, Country	Age/Sex	Underlying Condition/Risk Factor/Source	Time from Event to Empyema (Days)	Clinical Presentation	Comorbidities	Management Strategy	Antifungal Therapy	Outcome
Bonatti (2009), Austria [11]	58/F	Post-op hemorrhage (Lung Tx)	105	-	-	Percutaneous drainage	Fluconazole (P) * → Caspofungin, Voriconazole	Cured. Died of graft failure at 5 mos.
	59/M	Eso. perf./ Boerhaave syndrome	7	Sepsis	-	Eso. leak closure + decortication + CT- guided drainage	Fluconazole (P) * → Caspofungin	Cured.
Cascio (2010), Italy [12]	45/F	Eso. perf.	-	Fever, fatigue, dyspnea, constipation, nausea	7 months pregnant, heavy drinker	Chest tube + Dx Thoracotomy	Fluconazole → Voriconazole → Caspofungin	Cured. Died of MOF on the 42nd day
Srinivasnakshatri (2014), India [13]	11/F	PDA device closure/community-acquired	-	Abdominal pain, nausea	PDA (closed)	Thoracentesis (Dx) + ICD	Amphotericin B	Died.
Bukamur (2018), USA [14]	74/F	Eso. perf.	-	Nausea, vomiting, dysphagia	Schizophrenia, dementia, COPD, CVA	Chest tube, decortication + extensive pleural peel	Micafungin → Voriconazole	Cured.
Vrba (2022) [15], Czech Republic	69/M	Anastomosis dehiscence post-esophagectomy	11	Respiratory failure, septic shock	SCC esophagus, alcohol and tobacco abuse	Surgery + CT-guided drainage	Fluconazole	Cured. Discharged after 56 days.
Our Patient (2024), USA	70/M	B/L Lung Tx	81	Shortness of breath on exertion	IPF, OSA, HTN, DM, CAD (S/P PCI 2019)	Thoracocenteses (multiple)	Caspofungin → Anidulafungin → Voriconazole → Voriconazole + Micafungin	Cured.

* (P): drug used as prophylaxis. Post-op: Postoperative; Lung Tx: Lung transplant; Eso. perf.: Esophageal perforation; Eso. leak closure: Closure of esophageal leak; CT: Computed tomography; Dx: Diagnostic; ICD: Intercostal drainage; MOF: Multi-organ failure; PDA: Patent ductus arteriosus; COPD: Chronic obstructive pulmonary disease; CVA: Cerebrovascular accident; SCC: Squamous cell carcinoma; B/L: Bilateral; IPF: Idiopathic pulmonary fibrosis; OSA: Obstructive sleep apnea; HTN: Hypertension; DM: Diabetes mellitus; CAD: Coronary artery disease; S/P: Status post; PCI: Percutaneous coronary intervention.

## Data Availability

The original contributions presented in this study are included in the article. Further inquiries can be directed to the corresponding author.

## Abbreviations

The following abbreviations are used in this manuscript:

NAC	*Non-albicans Candida*
AST	American Society of Transplantation
ESCMID	European Society of Clinical Microbiology and Infectious Diseases
AFST	Antifungal Susceptibility Testing
ECMM	European Confederation of Medical Mycology
ISHAM	International Society for Human and Animal Mycology
ASM	American Society for Microbiology
SoR	Strength of Recommendation
QoE	Quality of Evidence
IPF	Idiopathic pulmonary fibrosis
BAL	Bronchoalveolar lavage
